# Oral lichen planus: clinical and histopathological considerations

**DOI:** 10.1016/S1808-8694(15)31102-2

**Published:** 2015-10-19

**Authors:** Fernando Augusto Cervantes Garcia de Sousa, Luiz Eduardo Blumer Rosa

**Affiliations:** 1 Master in Oral Biopathology, FOSJC/UNESP. Dental Surgeon; 2 Adjunct Professor of Oral Pathology, FOSJC/UNESP, Dental Surgeon

**Keywords:** diagnosis, literature review, lichen planus, mouth mucosa

## Abstract

Oral lichen planus is one of the most common dermatological diseases presenting in the oral cavity; the prevalence in the general population is 1% to 2%. Although relatively frequent, oral lichen planus is the target of much controversy, especially in relation to its potential for malignancy.

**Aim:**

This study aimed to make clinical and histopathological considerations regarding oral lichen planus to increase the level of knowledge about this condition among health professionals, underlining the importance of long-term follow-up of these patients.

**Conclusion:**

The possibility of this lesion to turn malignant justifies the importance of long term follow up for patients with such disease.

## INTRODUCTION

Lichen planus is a chronic inflammatory disease that affects the skin and mucosa. It is one of the most common dermatological conditions involving the oral cavity; its prevalence is 1% to 2% in the general population. There is a strong preference for the female sex^1^

Sousa & Rosa^2^ (2005) surveyed 79 oral lichen planus cases diagnosed between 1974 and 2003, and found that women are nearly four times more affected by this condition than men, and that white individuals are five and a half times more likely to develop this disease compared to other races.

These features, however, are among the few points of agreement about oral lichen planus; most of the remaining aspects are controversial, especially it's potential for malignant transformation. The aim of this paper, therefore, is to describe oral lichen planus clinically and histopathologically, and to help disseminate this information among health professionals; the importance of long-term follow-up of these patients is underlined.

## ETIOPATHOGENESIS

Although oral lichen planus was initially described in 1869, little is known about mechanisms by which the disease develops.

Sugerman et al.^3^ (2002) believe that specific and non-specific mechanisms may be involved in the etiopathogenesis of this condition. Specific mechanisms include antigen presentation by basement layer keratinocytes and cytotoxic T lymphocyte-caused death of antigen-specific keratinocytes, while non-specific mechanisms included mast cell degranulation and matrix metalloproteinase activation. These combined mechanisms appear to cause T lymphocytes accumulation in the lamina propria underlying the epithelium, as well as rupture of the basement membrane, intraepithelial T lymphocytes migration and keratinocyte apoptosis, all of which are characteristic of oral lichen planus. Furthermore, according to these authors, the chronic nature of this disease may be partly explained by deficient immunosuppression, mediated by the transforming growth factor-beta ^1^.

The factors that set this process in motion, however, have not been fully clarified. Still, stress, food such as tomatoes, citric fruit and seasoned dishes, dental procedures, systemic disease, alcohol abuse, and tobacco use in all its forms, have been associated with disease exacerbation periods.^4^ Recently, systemic diseases, especially those resulting from hepatitis C virus infection, have come under the spotlight.

Lodi et al.^5^ (2004) used the enzyme-linked immunosorbent assay (ELISA) test to investigate the presence of antibodies against the hepatitis C virus in 581 patients, 303 of which with a clinical and histopathological diagnosis of oral lichen planus, and 278 with no evidence of this disease (control group). Of 303 patients diagnosed with oral lichen planus, 58 (19.1%) were positive for the hepatitis C virus, compared to only nine (3.2%) in the control group. Furthermore, the authors reviewed the results of 24 similar studies done between 1994 and 2003, and found a statistically significant difference in the proportion of serum positive individuals for the hepatitis C virus among patients with oral lichen planus compared to controls.

The relation between oral lichen planus and the hepatitis C virus is not consistent; the prevalence of this virus in such patients varies widely from 0% to over 60%, depending on the country in which these studies were conducted. It is thought that such differences are mainly due to geographic discrepancies in the prevalence of this virus within the general population.^6^

Henderson et al.^7^ (2001) have questioned this hypothesis. The author assessed the oral health and the availability of dental treatment for hepatitis C virus infected-patients in the United Kingdom, and found clinical evidence of lichen planus in 20% of these patients. This was a much higher percentage than that found in the general population, in which the prevalence was not more than 1%. This finding came to the author's attention, as the prevalence of the hepatitis C virus is low; the literature shows that there is a directly proportional relation between the prevalence of the hepatitis C virus and oral lichen planus.

Cunha et al.^8^ (2005) noted similar findings in a study of 134 serum positive patients for the hepatitis C virus in Brazil. Although the prevalence of this virus is high in this country, the percentage of patients with lichen planus was 1.5%; there was, therefore, no statistically significant difference compared to the control group, in which 1.1% of patients presented signs of the disease.

A direct association between lichen planus and the hepatitis C virus cannot always be demonstrated. Mico-Llorens et al.^9^ (2004), for instance, found no changes in the oral mucosa of 100 patients infected with the hepatitis B or C or both viruses that participated in a study done by the Digestive System Pathology Unit of the “Príncipes de España de Bellvitge” Hospital in September and October 2000.

Romero et al.^10^ (2002), however, have underlined the need to investigate the presence of hepatitis C virus antibodies in all patients with oral lichen planus. These authors believe that the existence of clinical variants of the disease, in terms of its site and number of intra-oral lesions in hepatitis C virus-infected and non-infected patients, suggests that this virus has a significant role in the progression of lichen planus.

Psychological factors have recently been strongly associated with lichen planus, in particular high stress and anxiety levels. Although this association has been known for decades, difficulties in objectively measuring these variables has meant that only recently has the importance of anxiety and stress been widely recognized; these factors are now the target of numerous studies.

Soto-Araya et al.^11^ (2004) investigated the relation between psychological disorders -stress, anxiety and depression - and disease of the oral mucosa. The author applied psychological profile tests in 18 patients with recurrent aphthous stomatitis, nine with oral lichen planus, seven with burning mouth syndrome, and 20 with no apparent lesion. The results suggested a statistically significant relation between the presence of psychological changes and the diseases that were studied. The authors found that stress levels were highest in patients with recurrent aphthous stomatitis and oral lichen planus. Anxiety levels were highest in all three groups of patients compared with the control group. Evidence of depression was observed especially in patients with the burning mouth syndrome. These results led to the conclusion that there was a close relation between psychological alterations and certain disease that affect the mouth; it also highlighted the influence of psychic factors on oral health.

Koray et al.^12^ (2003) studied the relation between anxiety and levels of cortisol - the stress hormone - in the saliva of 40 patients with oral lichen planus. The analysis compared anxiety and salivary cortisol levels in these patients with the control group; the authors concluded that both levels were considerably higher in patients with oral lichen planus compared to those without this disease, further strengthening the ties between altered stress and anxiety levels and oral lichen planus. Ivanovski et al.^13^ (2005) has confirmed this conclusion after finding the same results in a basically similar study.

There are, therefore, major debates around the mechanisms by which lichen planus develops and why they occur. Although there is evidence that viral infections - especially those caused by the hepatitis C virus - and psychological disorder somatization, such as stress and anxiety, may be possible causes of this disease, information is lacking to definitively confirm these connections. Further debates have arisen about the possible malignant nature of oral lichen planus.

## CLINICAL FEATURES

Lichen planus is a frequent mucocutaneous disease that affects mostly women between the fifth and sixth decades of life.^2^^,^^4^^,^^14^, ^15^, ^16^ According to Laeijendecker et al.^17^ (2005), however, the disease may also affect individuals aged below 18 years; the clinical features are the same as those that present in older adults, but the prognosis is more benign.

Clinically, oral lichen planus has specific and clearly identifiable features,^18^ usually presenting in one of two main forms - the reticular and the erosive forms - although other forms are not rare.^14^ In fact, according to Mollaoglu^19^ (2000), four other forms were originally described: the papular, “plate-like”, bullous and atrophic forms.

The reticular form occurs more frequently and is characterized by white lacy streaks known as Wickham's striae, which generally are surrounded by discrete erythematous borders. Such feature may not be evident in certain sites, such as the dorsum of the tongue, where lesions present as keratotic plaques. The reticular form usually causes no symptoms; it involves the posterior jugal mucosa bilaterally. Other sites may be simultaneously involved, such as the upper and lateral surfaces of the tongue, the gums and the palate.^14^, ^15^^,^^19^

The erosive form is not as common as the reticular form, but it is more significant for patients, as the lesions are commonly symptomatic. Symptoms may range from discomfort to intensely painful episodes that interfere with chewing. Clinically, erosive lichen planus manifests as atrophic and erythematous areas frequently surrounded by radiating thin striae. In certain cases, the epithelium may separate if erosion is severe, resulting in a relatively rare form of the disease known as bullous lichen planus.^14^, ^15^

At times atrophy and ulceration is confined to the gingival mucosa, in a reaction pattern named desquamative gingivitis. Such cases should be biopsied for immunofluorescence and optic microscopy of perilesional tissue, as benign mucous membrane pemphigoid and pemphigus vulgaris may present similarly.^15^

Mignogna et al.^20^ (2005) assessed 700 patients with oral lichen planus and reported that 48% presented gingival involvement, and that lesions occurred only in the gums in 7.4% of the sample patients. The authors also found that 20% of cases of malignant transformation involved the gingival mucosa, further reinforcing the importance of histopathology in the final diagnosis of this disease.

Patients with oral lichen planus frequently present one or more extraoral lesions. Eisen^4^ (2002), for instance, mentions that about 25% of women with this disease also present concomitant vulvovaginal mucosal involvement, and that about 15% of all patients with this disease also have skin lesions. According to Neville et al.^15^ (2004), skin lesions have been classified as polygonal, pruritic, purple papules. These usually affect the flexor surfaces of extremities. Scoriation may not be visible, although lesions may be irritative; they are painful when scratched. Careful examination of the surface of papules reveals fine reticular white lacy streaks (Wickham's striae). Other extraoral sites are the nails, the scalp, the penile glans and the esophageal mucosa.

Skin lesions facilitate considerably the diagnosis of lichen planus. However, with the exception of the reticular form, which has pathognomonic features, most cases are diagnosed histopathologically. A differential diagnosis should be made between erosive lichen planus and epidermoid carcinoma, discoid lupus erythematosus, chronic candidiasis, benign mucous membrane pemphigoid, morsicatio buccarum, lichenoid reaction to amalgam or to drugs, graft versus host disease, and erythema multiforme. Lichen planus reticularis should, in some cases, be differentiated from leukoplasia.^14^

## DIAGNOSIS AND TREATMENT

The typical clinical features of oral lichen planus are usually sufficient for the diagnosis of this condition. Still, a biopsy for histopathology is recommended to confirm the clinical diagnosis and mainly to exclude epithelial atypia and signs of malignancy.^18^

**Figure 1:**
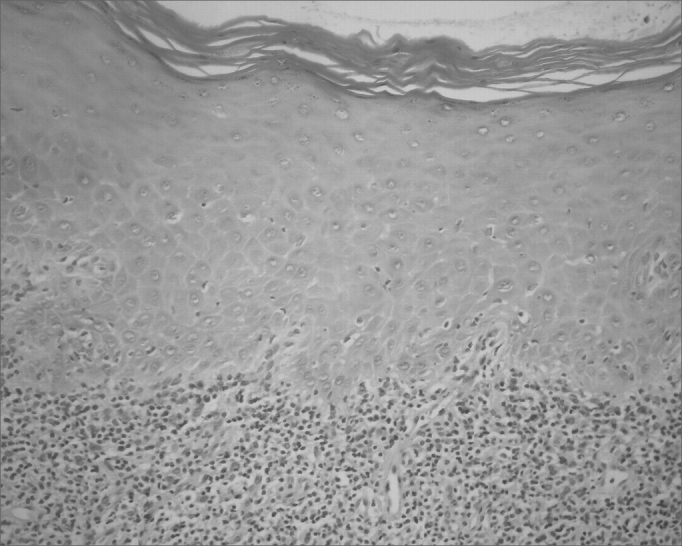
Lichen planus. Histology section showing epithelial hyper-parakeratosis, mild acanthosis and basement layer lichenification; also present is a layered marked lymphocyte infiltrate immediately underlying the epithelium. H/E – 400x.

**Figure 2:**
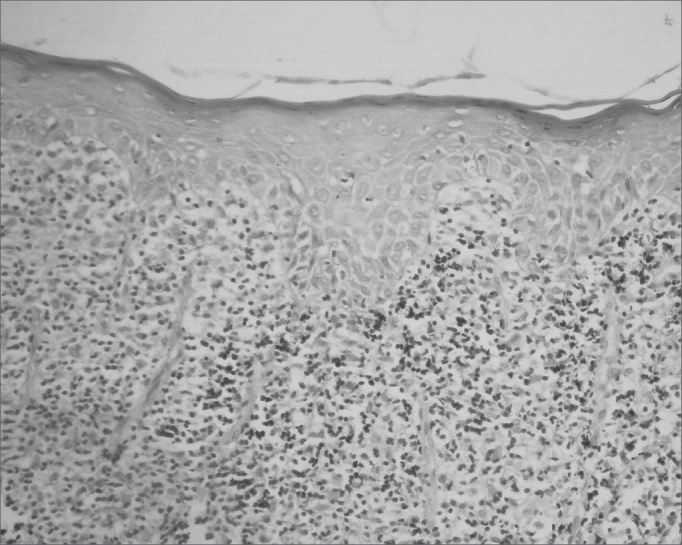
Lichen planus. Note areas of epithelial atrophy, and a layered marked lymphocyte infiltrate in the lamina propria immediately underlying the epithelium. H/E – 400x.

**Figure 3:**
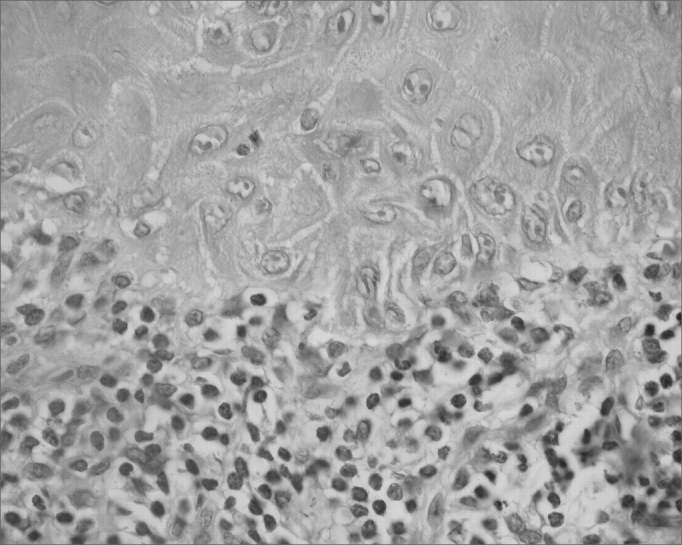
Lichen planus. The epithelium shows large keratinocytes containing prominent nuclei and basement layer liquefaction. Decreased melanin pigmentation in the lamina propria, and a layered marked lymphocyte infiltrate immediately underlying the epithelium. H/E – 400x.

**Figure 4:**
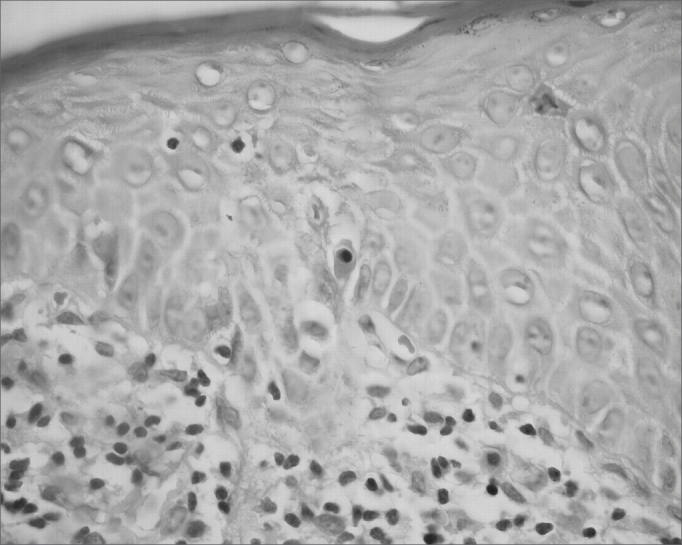
Lichen planus. The epithelium shows basement layer liquefaction. Civatte body (center). H/E – 400x.

The classical histopathological findings in oral lichen planus are: lichenification of the basement layer, followed by a marked layered lymphocytic infiltrate immediately underlying the epithelium; the presence of numerous eosinophilic colloid bodies along the epithelial-connective tissue interface (Civatte bodies); absent, hyperplasic or, more frequently, sawtooth-shaped interpapillary ridges; variable thickness of the spinous layer; and variable degrees of ortho or parakeratosis.^14^, ^15^^,^^21^, ^22^ (Figure 1, Figure 2, Figure 3, Figure 4)

Other conditions may, however, present similar histopathological findings to those of oral lichen planus; these include lichenoid reactions, lupus erythematosus, leukoplasia, erythroleukoplasia, and proliferative verrucous leukoplakia (PVL). In its initial phase, PVL presents clinical and histopathological findings that may easily be mistaken for those found in lichen planus. PVL, however, frequently reveals varied degrees of epithelial atrophy and high malignant transformation rates.^23^

For this reason, Kolde et al.^24^ (2003) have suggested using routine direct immunofluorescence in the diagnosis of oral lichen planus, particularly when other autoimmune diseases are included in the differential diagnosis. According to Regezi & Sciubba^22^ (2000), direct immunofluorescence reveals the presence of fibrinogen along the basement membrane zone in 90% to 100% of cases. Although immunoglobulins and complement factors may also be found, they are much less common that fibrinogen deposits.

The histopathological diagnosis of oral lichen planus requires careful attention. Van der Meij & Van der Waal^25^ (2003), for instance, found that in 42% of cases in which there was full agreement on the clinical diagnosis of this disease, there was no consensus in the histopathological diagnosis. Furthermore, in 50% of cases in which a histopathological consensus was achieved, clinical agreement was lacking.

Some authors have suggested that erosive and reticular lichen planus should be considered in separate. According to Karatsaidis et al.^26^ (2003), the considerably increased cell proliferation in erosive lichen planus, compared to the reticular form, indicates that the erosive form is more active; this underlies the importance of studying each form separately. Seoane et al.^27^ (2004) have reinforced this conclusion stating that the reticular and erosive forms have different biological behaviors.

Finally, Lukac et al.^28^ (2006) assessed serum concentrations of antibodies against desmoglein-1 and −3 by using the ELISA test in 32 patients with erosive lichen planus, in 25 patients with the reticular form, in 13 patients with recurrent aphthous stomatitis, and in 50 patients with no clinical signs. These authors found that differences in the serum concentration of antibodies against desmoglen-1 and −3, indicating that the etiopathogenic mechanisms of erosive and reticular lichen planus are dissimilar.

The integrity of margins is a further issue in the histopathological diagnosis of oral lichen planus due to its malignant potential. Redahan et al.^29^ (2005) have sated that about 50% of biopsies show compromised margins, suggesting that examination of the adjacent tissues should be part of any monitoring protocol for this disease.

Having confirmed the diagnosis, therapy aims to relieve symptoms, given that curing the disease is not always possible. According to Regezi & Sciubba^22^ (2000), corticosteroids are the drugs of choice in the treatment of lichen planus, due to their ability to modulate the inflammatory and immunological responses. Topical use and local injection of steroids have been used successfully for controlling lichen planus. Systemic steroids may be used for severe cases. Adding antifungal drugs improves clinical results, apparently by eliminating Candida albicans, which grows secondarily in tissues affected by lichen planus. Antifungal drugs also prevent corticosteroid-associated fungal growth.

Other drugs have been used in the treatment of lichen planus with excellent results; these include immunosuppressant such as cyclosporin^30^ and tacrolimus.^31^, ^32^ Theoretically, these drugs may increase the likelihood of malignant transformation, as they not only affect the immune systems, but act directly on cells.^23^

Becker et al.^33^ (2006), for instance, believe that the relation between topical use of tacrolimus and the development of epidermoid carcinoma in patients with oral lichen planus is not limited only to immune suppression. These authors have suggested that tacrolimus appears to interfere on certain important intracellular signaling pathways, especially those related with the p53 protein; this protein is also altered in a various types of cancer.

## MALIGNANT POTENTIAL

In the past decades many papers have suggested that patients with oral lichen planus are at an increased risk of developing cancer, which led the World Health Organization to classify this disease as a premalignant condition. The association between oral lichen planus and epidermoid carcinoma, however, is still polemic; many authors believe that there are not enough data to prove this association. For these authors, most of the cases of malignant transformation could not be considered as such, as there were already alterations that suggested malignancy upon the initial diagnosis of lichen planus. Nevertheless, many papers highlight the malignant potential of this disease.^21^^,^^34^

Eisen^4^ (2002) studied the malignant transformation potential of oral lichen planus, as well as its clinical features and possible relation with systemic alterations. This author monitored 723 patients with oral lichen planus for a period between six months and eight years. The study found that epidermoid carcinoma developed in sites with a previous diagnosis of lichen planus in 0.8% of patients. The author then suggested that periodic monitoring of these patients was essential, given the increased risk of developing epidermoid carcinoma in sites involved by oral lichen planus.

Gandolfo et al.^35^ (2004) undertook a similar study that evaluated 402 patients with oral lichen planus that had been diagnosed between January 1988 and July 1999; these patients were monitored periodically until February 2001. During the follow-up period, two male (1.3%) and seven female (2.8%) patients developed epidermoid carcinoma; the risk of malignant transformation was higher among women compared to men. Additionally, patients infected by the hepatitis C virus had a threefold increase in the chance of developing oral cancer compared to non-infected patients. These results not only reinforce suspicions about the malignant transformation potential of lichen planus, but also have focused attention of the role of the hepatitis C virus in this process.

Xue et al.^16^ (2005) investigated the clinical features of oral lichen planus in 674 patients of the Stomatology Unit, Wuhan university, China, between 1963 and 2003. About 0.65% of the 674 patients developed epidermoid carcinoma in sites with a previous diagnosis of erosive or atrophic lichen planus, suggesting that both forms of the disease increase the risk of malignant transformation more than the reticular form. The authors also called attention to the need to monitor patients with lichen planus for many years, as there was one case in which malignant transformation occurred 21 years after the initial diagnosis of lichen planus.

The results above confirm those of Lanfranchi-Tizeira et al.^36^ (2003). These authors evaluated 719 oral lichen planus cases diagnosed at the Oral Clinical and Pathology Unit II, Dentistry School, Buenos Aires University, Argentina, between 1991 and 1997, and found that all the 32 cases of malignant transformation (6,51%) occurred in atypical forms of lichen planus, such as presentation in the plaque, the erosive and the atrophic form.

Why such transformation occurs remains unclear. Mignogna et al.^37^ (2004) has suggested that currently there is sufficient evidence demonstrating that chronic inflammation, which is the case of oral lichen planus, generates a cytokine-based microenvironment that affects cell survival, growth, proliferation and differentiation; this may consequently contribute to cancer initiation, promotion and progression.

Other authors, however, believe that transformation is favored by an altered expression of apoptosis-regulating proteins, such as the p53 protein. According to Neppelberg et al.^38^ (2001), the number of epithelial cells undergoing apoptosis is considerably increased in sites affected by lichen planus, compared to the normal epithelium.

Valente et al.^39^ (2001) analyzed p53 protein overexpression in the biopsies of 28 patients with oral lichen planus done periodically during 96 months. No dysplasia was seen in 15 of these patients across the study period (group 1). In seven patients there was synchronism between the diagnosis of lichen planus and the development of epidermoid carcinoma (group 2); in the remaining patients, this progression was seen months or years later (group 3). The percentage of p53-positive epithelial cells was considerably higher in groups 2 and 3 compared to group 1. The cell proliferation rate, evaluated by the immunohistochemical expression of the MIB-1 protein, was not statistically different among the groups. Although no conclusion is yet possible about the molecular pathways that cause oral lichen planus to undergo malignant transformation, results suggest that an immunohistochemical assessment of p53 expression may become a useful tool for selecting those cases at a higher risk for malignancy.

Lee et al.^40^ (2005) investigated the expression of the p53 protein and the proliferating cell nuclear antigen (PCNA) in oral lichen planus and its relation with the clinical behavior of the disease and the habits of patients. The authors used immunohistochemical tests to evaluate the expression of those proteins in 56 patients, which were then compared with samples of normal, hyperkeratotic and dysplasic oral mucosa, and with epidermoid carcinoma samples. PCNA and p53 expression in oral lichen planus was similar to that found in hyperkeratotic mucosa, but higher than that seen in normal mucosa, and lower than that found in dysplasic mucosa and in the epidermoid carcinoma. There was, however, no significant correlation between the expression of both proteins and any clinical feature of the disease. But the expression of p53 was higher in patients with the habit of chewing a mixture of betel nuts with tobacco and slacked lime, while the expression of PCNA was higher in the atrophic forms of the disease compared to the hypertrophic forms. Patients with the atrophic forms of lichen planus and the habit described above had considerably higher expression of these proteins, similar to that found in dysplasic mucosa and in the epidermoid carcinoma. According to the authors, these results confirm the premalignant nature of oral lichen planus; they suggest that the atrophic forms of this disease have a higher malignant potential, especially when associated with the abovementioned habit.

Bascones et al.^41^ (2005) investigated the influence of apoptosis and cell cycle interruption mechanisms in the malignant transformation process of oral lichen planus. They assessed the apoptotic rate by the terminal deoxyribonucleotidyl transferase mediated dUTP nick end labelling (TUNEL) method and evaluated the immunohistochemical expression of bax, caspase-3 and p21 proteins in oral mucosa samples of 32 patients with oral lichen planus. The authors concluded that a low epithelial cell response to apoptosis and cell cycle interruption mechanisms could cause malignant transformation.

Although many studies have demonstrated the premalignant nature of oral lichen planus, many other have stated the opposite. Sousa et al.^42^ (2005), for instance, found no connection between oral lichen planus and the epidermoid carcinoma when using criteria such as sex, race, age and site of increased disease prevalence; this suggests that the profile of lichen planus patients differs considerably from that observed in patients with the epidermoid carcinoma.

Various authors believe that most of the cases of malignant transformation described in the literature should not be considered as such, as they show a range of epithelial atypia on the initial diagnosis, which would define a condition with distinct histopathological features named lichenoid dysplasia. According to Lodi et al.^23^ (2005), inflammation present in oral lichen planus may cause cell alterations similar to those seen in epithelial atypia, making it even more difficult to differentiate from lichenoid dysplasia.

Recent papers have recast the idea that lichen planus and lichenoid dysplasia should be considered as two different entities. The presence of epithelial atypia is currently believed to be the factor that classifies a lesion as having malignant potential; thus, lichenoid dysplasia, rather than lichen planus, should be classified as premalignant. The possibility of malignant transformation is a reflection of many intrinsic molecular alterations within cells, all of which are found in lichenoid dysplasia.^43^

Kim et al.^44^ (2001) assessed the malignant potential of oral lichen planus by comparing the degree of genetic instability in clinically cured cases and in those that had undergone malignant transformation. These authors concluded that lichenoid dysplasia should be seen as a high-risk premalignant lesion, and that chromosome nine monossomia may have an important role in the malignant transformation of this lesion.

Van der Meij et al.^45^ (2003) conducted a prospective study of 62 patients with oral lichen planus and 111 patients with lichenoid dysplasia during 6.6 to 72 months. Three of 173 patients (1.7%), progressed to epidermoid carcinoma; malignant transformation in all cases developed from lichenoid dysplasia. The authors suggested that further prospective studies with larger series were needed before reaching a final conclusion about the malignant potential of oral lichen planus and lichenoid dysplasia. They also underlined that, whether lichen planus is or not considered as a premalignant lesion, the mere suspicion of such a possibility justified long-term monitoring of these patients.

Mattsson et al.^46^ (2002) have stated that although there may be enough indications that lichen planus has malignant potential, its low rate makes periodic specialist monitoring of these patients financially unfeasible. Still, it is important that health professionals know the early signs of mouth cancer so that it may be detected in routine care. Additionally, patients should be instructed to inform any change in their condition.

## CONCLUSION

Regardless of the polemic around oral lichen planus, which should be restricted to academic levels, in practice the mere possibility of malignant transformation justifies long-term monitoring of patients with this disease. This becomes even more evident given the difficulties in making the clinical and histopathological diagnosis of this condition. Care should be taken when informing patients about these issues to avoid excessive worry that would only worsen the clinical picture.
